# Prostate cancer treated with reduced-volume intensity-modulated radiation therapy: Report on the 5-year outcome of a prospective series

**DOI:** 10.1097/MD.0000000000009450

**Published:** 2017-12-29

**Authors:** Hua-Chun Luo, Zhi-Chao Fu, Hui-Hua Cheng, Yong Lei, Shao-Guang Liao, Jing Feng, Qin Yin, Qun-Hua Chen, Gui-Shan Lin, Jin-Feng Zhu, Jian-Feng Xu, Wang Dian

**Affiliations:** a Department of Radiation Oncology, Fujian medical university affiliated Fuzhou General Hospital; b Department of Medical, Fuzhou General Hospital of Nanjing Command PLA, Fuzhou; c Department of Medicine, Longyan Hospital of Traditional Chinese Medicine, Longyan; d Department of Radiation Oncology, Fujian Province Hospital, Fuzhou; e Department of Urology, Jinjiang Hospital, Quanzhou, China; f Department of Radiation Oncology, Rush University Medical Center, Chicago, IL.

**Keywords:** clinical target volume, prognosis, prostate cancer, radiotherapy

## Abstract

How to define a clinical target volume (CTV) as small as possible for prostate cancer to reduce the dose received by normal organs is an interesting study. We conduct a research to analyze the clinical efficacy of intensity modulated radiotherapy (IMRT) using reduced CTV in the treatment of prostate cancer. From January 2006 to June 2010, 78 patients with prostate cancer were treated with IMRT according to this institutional protocol. Of them, 18 had stage II tumors, 39 had stage III tumors, and 21 had stage IVa tumors. Clinical outcomes included overall survival, biochemical recurrence, recurrence-free survival, and acute and chronic injuries caused by radiotherapy. Risk factors were evaluated using the Cox regression model. As of December 31, 2014, all patients completed radiotherapy as planned. Myelosuppression was mostly grade 1, acute urinary injury was mostly grades 1 and 2, and intestinal injury was mostly grade 1. The 5-year follow-up rate was 91.0%. The overall, progression-free, biochemical recurrence-free, and distant metastasis-free survival rates were 82.1%, 79.4%, 84.6%, and 94.9%, respectively. Tumor volumes defined by small target volumes and Radiation Therapy Oncology Group were 274.21 ± 92.64 and 600.68 ± 113.72, respectively, representing a significant difference (*P* < .05). Age, prostate-specific antigen level, eastern cooperative oncology Group score, Gleason score, and volume of CTV were independent risk factors for mortality and disease progression. Our findings indicated that IMRT with reduced CTV have less acute and chronic injuries caused by radiation, particularly grade 3 or higher urinary and intestinal injuries, while ensuring survival benefits and protecting the hematopoietic function.

## 1 Introduction

As the most commonly diagnosed malignant tumor in American men, prostate cancer is also increasingly diagnosed in China.^[1,2]^ As a primary treatment, intensity modulated radiotherapy (IMRT) plays an important role in improving survival in patients with prostate cancer.^[3,4]^ Our previous 8-year follow-up of patients with locally advanced prostate cancer who received IMRT combined with hormonal therapy revealed that urinary and bowel symptoms were the main factors affecting quality of life,^[5]^ which may be related to an increase in the high-dose irradiated volume in the bladder and rectum or poor dose uniformity in the prostate target volume. The use of IMRT to increase irradiated volume may increase the risk of a second tumor.^[6]^ Therefore, a key challenge for external beam radiotherapy of prostate cancer should be how to define a clinical target volume (CTV) as small as possible to safely increase tumor irradiation dose while reducing the dose received by normal organs, in order to decrease long-term radiation side effects. In this study, a prostate cancer IMRT plan was designed to provide reduced CTV s relative to Radiation Therapy Oncology Group (RTOG)-defined target volumes, and 5-year biochemical recurrence rate, progression-free survival, overall survival, and side effects were followed, in order to provide a reference for defining the external irradiation target volumes in prostate cancer.

## 2 Methods

### 2.1 General information

This study was approved by the Ethics Committee of Fuzhou General Hospital. Each patient signed a treatment consent 1 week before the treatment. Inclusion criteria included diagnosis of prostate cancer confirmed by histopathology, eastern cooperative oncology Group (ECOG) score ≤ 2, never received androgen deprivation therapy or brachytherapy and proton therapy before, no distant organ metastasis revealed by imaging, and no previous history of cancer. From January 2006 to June 2010, 127 treatment-naive patients with pathologically confirmed prostate cancer received chest computed tomography (CT), color Doppler ultrasound of digestive system, pelvic magnetic resonance imaging (MRI), and whole-body bone scintigraphy. Of them, 47 did not meet the inclusion criteria (8 with bone metastasis) and 2 did not complete radiotherapy. At last, 78 prostate cancer patients without distant metastases were enrolled in the study. General information of enrolled patients is summarized in Table 1.

**Table 1:**
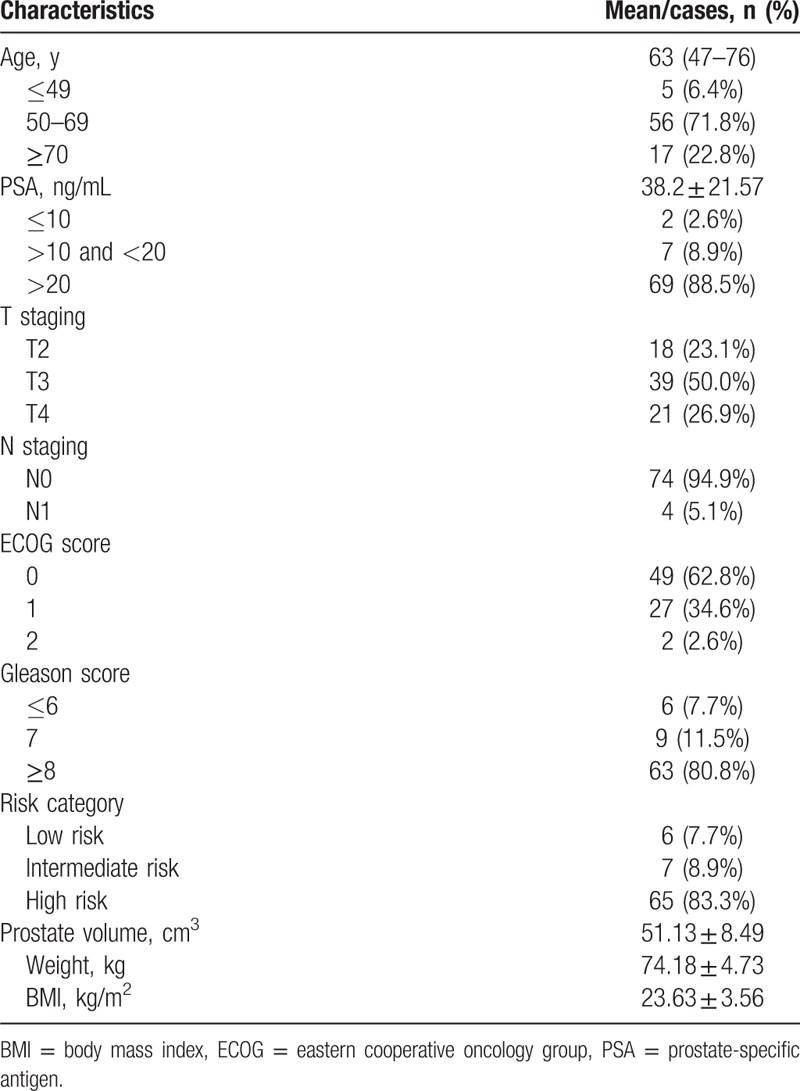
Characteristics of study patients.

### 2.2 IMRT procedure

All subjects were immobilized with phantoms and were instructed to empty their bladder and rectum 1 h before localization, take 1000 mL of diluted intestinal contrast agent orally, fill their bladder, and lie in supine position. CT simulation was performed with a scan range from the lower border of L4 to 10 cm below the end of the ischium. CT images were transferred to the treatment planning system (varian CMS4.0 planning system). Localization CT and MR images were fused using Oncentra Master Plan Version 3.3 (Nucletron.BV).

### 2.3 IMRT with reduced CTV were defined and delineated as follows

#### 2.3.1 Definition of target volumes in prostate and seminal vesicles

Low-risk radiotherapy target volumes included prostate only, and intermediate- to high-risk radiotherapy target volumes included prostate and seminal vesicles. Prostate target volumes included all tissues between the base and apex of prostate (0.5 cm above the bulbourethral or upper border of the crus penis), as well as all calcified lesions (if any); and seminal vesicles included tissues within 2 to 2.5 cm next to the prostate cancer. The planning target volume (PTV) was defined by expanding the CTV by 0.5 cm posteriorly and by 1 cm in the other directions. A total dose of 72.6 Gy was prescribed and given at 2.2 Gy/f for 33 times.

#### 2.3.2 Definition of target volumes in pelvic lymph node drainage area

**Figure 1:**
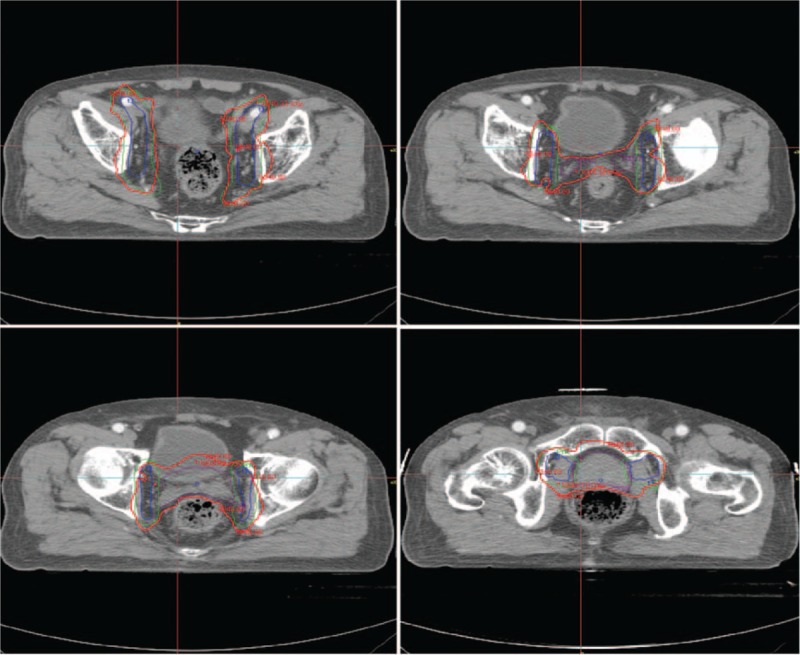
Prostate cancer (T3N0M0)-delineation of target volumes (red line, planning target volume [PTV] for prostate tumor volume; blue line, PTV for pelvic lymph node drainage area).

A short-axis diameter of pelvic lymph nodes of >1 cm in MRI T2 images was considered to meet clinical diagnostic criteria. For pelvic lymph nodes that did not meet the diagnostic criteria or were not visible, decision whether to perform prophylactic irradiation of the obturator lymph node drainage area was made according to the Roach formula; for positive obturator lymph nodes, the drainage area included obturator, internal and external iliac, and presacral lymph node drainage areas; and for positive internal and external iliac and/or presacral lymph nodes and/or positive common iliac lymph nodes, the drainage area included the whole pelvic lymph node drainage area. There was a case for the prostate cancer with T3N0M0 using IMRT-reduced CTV (see Fig. 1).

For the obturator lymph nodes, the target volume was delineated up to the upper margin of the pubic symphysis; for the external iliac lymph nodes, it was delineated up to the femoral head layer revealed by the localization CT; and for the presacral lymph nodes, it was delineated up to the piriformis layer. PTV of the pelvic lymph node drainage area was defined by expanding the CTV by 0.5 cm in the cephalocaudal direction and by 0.8 cm in the other directions. A total dose of 50.4 Gy was prescribed and given to PTV at 1.8 Gy/f for 28 times. A total dose of 66 Gy was prescribed and given to pelvic lymph nodes at 2.0 Gy/f for 33 times. Normal tissues and organs were delineated according to RTOG criteria. It was required that 95% of the PTV received more than 100% of the prescribed irradiation dose: V70 ≤ 25% for rectum and bladder, V50 ≤ 5% for both femoral heads, and V70 ≤ 25% for pubis.

### 2.4 Hormonal therapy

Medium-risk and high-risk groups (T > T2b and/or prostate-specific antigen [PSA] ≥ 10 ng/mL and/or Gleason ≥ 7) underwent androgen deprivation with 50 mg of oral Casodex once daily and 3.6 mg of Zoladex via subcutaneous injection every 28 days for 30 months.

### 2.5 Follow-up

All patients received routine blood tests and liver and kidney function tests weekly during treatment, and were followed up every 3 months during the first 2 years after the end of treatment, then every 6 months for another 2 years, and thereafter annually. During the follow-up visits, laboratory tests of blood, liver and kidney functions, PSA, and testosterone, and imaging tests, including chest X-ray, color Doppler ultrasound of digestive system, and pelvic magnetic resonance spectroscopic imaging were performed. Acute and delayed toxicity were assessed according to the RTOG toxicity criteria.^[7]^ Biochemical recurrence was defined as an increase in the lowest PSA value by 2 ng/mL (nadir + 2 ng/mL) after radiotherapy.^[8]^

### 2.6 Statistical methods

All data were analyzed using SPSS software [v.13.0; Chicago, IL, USA. (formerly SPSS Inc.)]. Count data were expressed as mean ± standard deviation. Local control rate, progression-free survival, time to biochemical recurrence, and overall survival were calculated using the Kaplan–Meier method. Differences in survival were evaluated by the Log-rank test. Multivariate analysis of survival was performed using the Cox proportional hazards regression model. *P* < .05 was considered statistically significant.

## 3 Results

### 3.1 Treatment outcome

**Figure 2:**
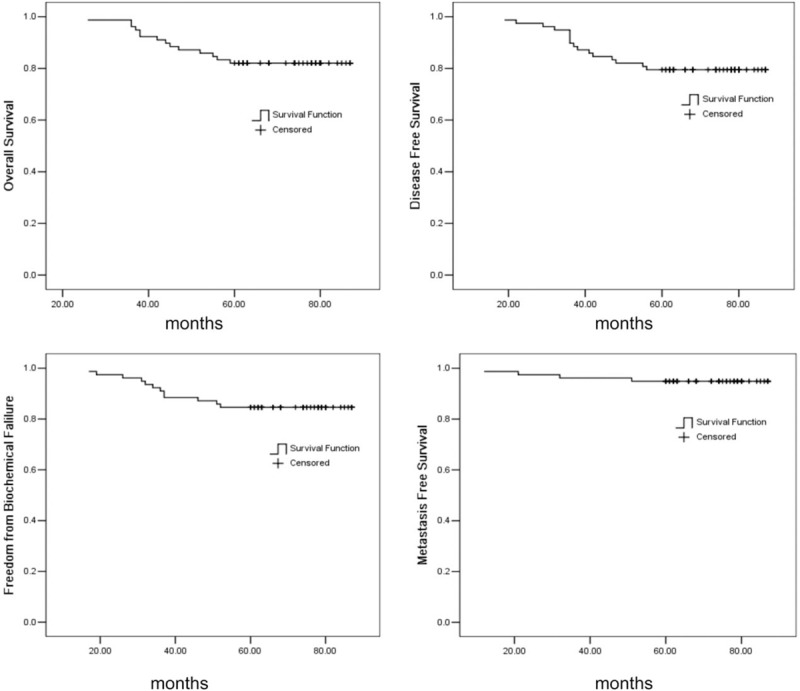
Kaplan–Meier estimate of (A) overall survival, (B) disease-free survival, (C) freedom from biochemical, and (D) metastasis-free survival.

As of December 31, 2014, the patients were followed up for 11.5 to 87.6 months, the median follow-up was 64.2 months, and 6 patients were lost to follow-up, with a follow-up rate of 91.0%. Fourteen patients died, of which 9 were due to intercurrent complications and 5 to prostate cancer, with a 5-year overall survival rate of 82.1%; 16 patients had disease progression, with a 5-year progression-free survival rate of 79.4%; 12 patients had biochemical recurrence, with a 5-year biochemical recurrence-free survival rate of 84.6%; and 4 patients had distant metastases, with 5-year distant metastasis-free survival rate of 94.9% (Fig. 2).

In the 12 patients with biochemical recurrence, the median time to PSA progression was 29 months (16–57 months). Among them, 2 patients had stage III tumors, 4 had T4N0 tumors, and 6 had T4N1M0 tumors; 5 and 7 patients had PSA ≤ 20 ng/mL and >20 ng/mL, respectively; and 2, 7, and 3 patients had an ECOG score of 0, 1, and, 2, respectively.

In the 4 patients with distant metastasis, the median follow-up time was 23 months (9–62 months). Among them, no patients had stage III tumors, 1 had T4N0 tumors, and 3 had T4N1M0 tumors; 2 patients had PSA ≤ 20 ng/mL and 2 patients > 20 ng/mL; and 1, 2, and 1 patients had an ECOG score of 0, 1, and, 2, respectively.

### 3.2 Tumor volumes defined by small target volumes and RTOG

All 78 patients had target volumes defined according to the above-mentioned principles (small target volumes) and the RTOG criteria before treatment, respectively. For the small target volumes, D_max_, D_100_, D_95_, and median dose were 7267.68 ± 573.69 (cGy), 5335.27 ± 467.81 (cGy), 6056.42 ± 510.73 (cGy), and 6461.83 ± 528.86 (cGy), respectively; Meanwhile, for the RTOG-defined target volumes, D_max_, D_100_, D_95_, and median dose were 7018.30 ± 596.97 (cGy), 5428.14 ± 436.29 (cGy), 6259.48 ± 530.84 (cGy), and 6529.81 ± 542.36 (cGy), respectively; there were no significant difference between the 2 target volumes. Tumor volume defined by small target volumes was 274.21 ± 92.64 (mL), which significantly lower than 600.68 ± 113.72 (mL), that defined by RTOG (Table 2).

**Table 2:**
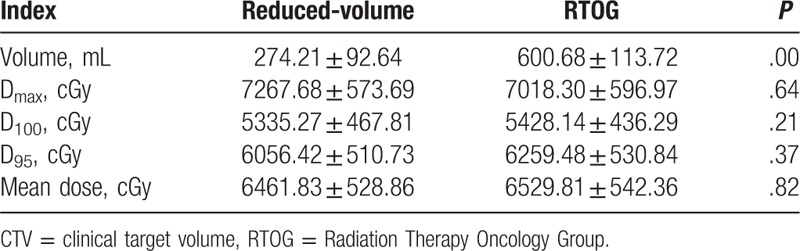
Differences of delineation of CTV between reduced-volume and RTOG.

The V70, V60, and V50 of rectum in reduced-volume were lower than RTOG; however, there were no significant difference between them (*P* > .05), the result was the same to the V50 of Femoral heads (*P* > .05). The V60, V50 of rectum and V50 of small bowel in reduced-volume were lower than RTOG, there were significant difference between them (*P* < .05) (see Table 3).

**Table 3:**
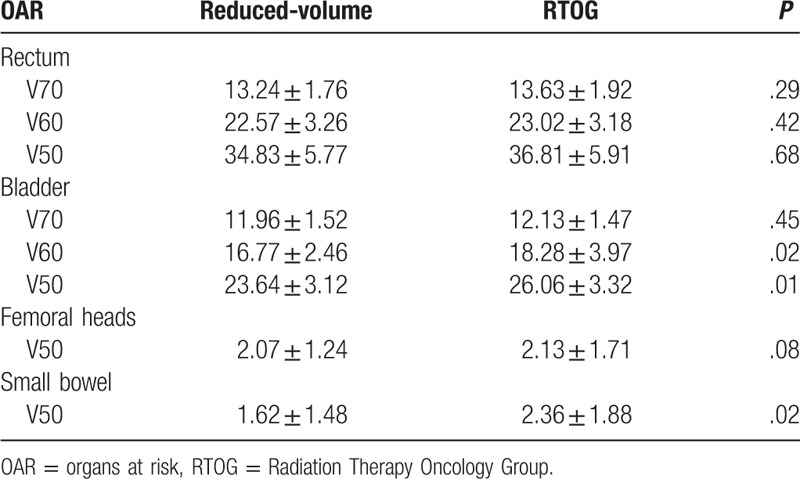
Difference dose to OAR between reduced-volume and RTOG (x ± S, %).

### 3.3 Acute and chronic radiation injuries

All 78 patients were able to tolerate the small target volume radiotherapy, and all completed radiotherapy as planned. During the 5 years of follow-up, no patients had grade 3 or higher leukopenia or decreased hemoglobin; leukopenia and decreased hemoglobin consisted mainly of grade 1 myelosuppression. Four patients experienced grade 3 or higher acute urinary symptoms, including 3 with grade 3 and 1 with grade 4, which recurred after cystoscopic electrocautery; The grades 1 and 2 incidence of acute urinary injury was 89.7%, the incidence of chronic urinary injury was 66.7%, 65.3%, 53.8%, 51.2%, 43.6%, and 50% at the time points of 3, 6, 12, 24, 48, and 60 months follow-up, respectively. Three patients experienced grade 3 acute intestinal symptoms, and most were grade 1 injury (including acute and chronic injuries), the incidence of grade 1 intestinal symptoms was 62.8%, 64.1%, 70.5%, 67.9%, 71.8%, 85.9%, and 91.0%, respectively. Results are shown in Table 4.

**Table 4:**
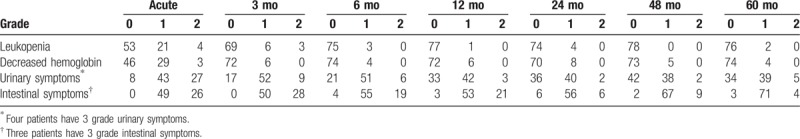
Acute and chronic radiation injuries.

### 3.4 Prognostic factors

Age, PSA level, tumor and node and metastasis stage, ECOG score, and Gleason score were independent risk factors for mortality, with an hazard ratio (HR) of 2.35 (1.03–5.80), 2.70 (2.42–5.06), 2.26 (1.32–4.18), 1.52 (1.13 3.26), and 1.89 (1.08–3.14), respectively. Age, PSA level, ECOG score, and Gleason score were risk factors for disease progression, with an HR of 2.64 (0.97–6.32), 2.93 (1.45–5.84), 1.26 (0.79–5.15), and 1.38 (1.32–3.31), respectively. Results are shown in Tables 5 and 6.

**Table 5:**
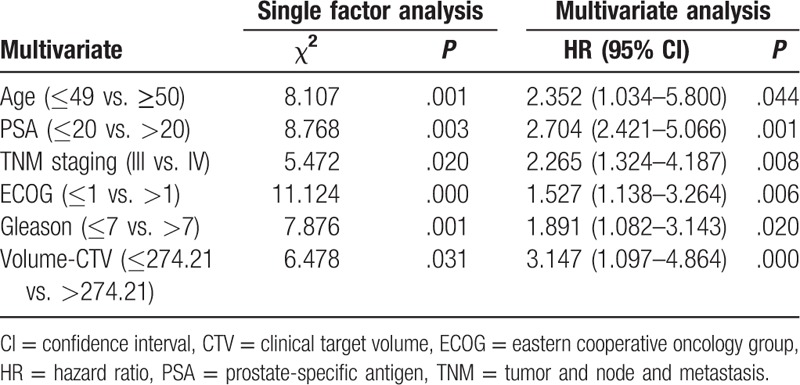
Cox single factor and multivariate regression analysis of overall survival.

**Table 6:**
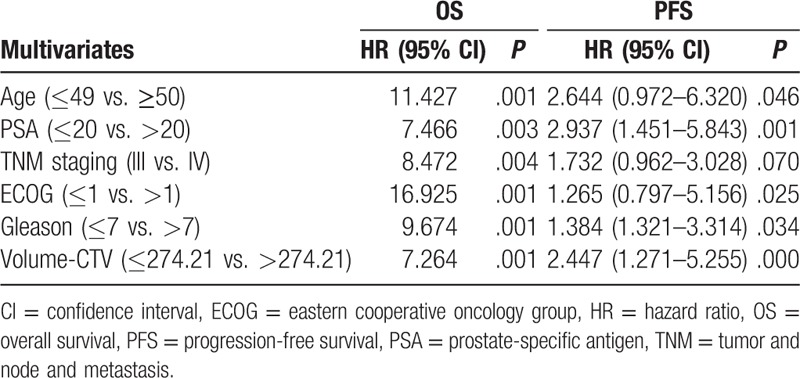
Cox single factor and multivariate regression analysis of PFS.

## 4 Discussion

Radiotherapy can improve disease progression-free survival in patients with locally advanced prostate cancer.^[9]^ Retrospective analysis of 1474 patients with locally advanced prostate cancer found that radiotherapy provided a better long-term survival,^[10]^ which was similar to the findings of our previous study.^[4]^ Small target volume IMRT combined with hormonal therapy provided 5-year overall and progression-free survival rates of 82.1% and 79.4%, respectively; the clinical overall survival was not significantly different from that in previous studies. Brachytherapy combined with external irradiation provided a 7-year biochemical recurrence-free survival rate of 57% in patients with locally advanced prostate cancer,^[11]^ and image-guided proton therapy provided a 5-year biochemical recurrence-free survival rate of 76%.^[12]^ The biochemical recurrence-free survival rate was 84.6% in this study, which may be related to the small sample size, and bias in age, PSA level, and other factors.

The application of IMRT decreased the average volume of rectum wall receiving 70 Gy (V70Gy) from 10–20% to 4.5–7.5%.^[13,14]^ In 260 patients with prostate cancer treated with IG-IMRT by Wortel et al,^[15]^ the median dose received by the anorectum and bladder was 34.4 and 33.1 Gy, respectively, which were significantly lower than those in this study. However, only 50% (n = 130) of patients enrolled in that study had T3 or higher tumors (50% vs. 78.1%). Bladder tolerance dose was significantly lower than the dose received by the bladder in high-dose radiation (86.4 Gy); and less clinical urinary symptoms were observed while survival was ensured; it requires further study to clarify whether these differences are related to the physical difference of oriental people.^[16]^ In the present study, dose received by bilateral femoral heads was lower than that in previous studies.^[15,16]^ Therefore, no patients had grade 3 or higher leukopenia or decreased hemoglobin during the entire follow-up, and myelosuppression was mostly grade 1. Small target volume IMRT can better reserve hematopoietic function in patients.

Acute or chronic injury caused by radiotherapy is proportional to radiation dose to organs at risk. However, external radiation dose was able to improve the local control rate and long-term survival in patients with prostate cancer.^[17]^ The prescribed dose was 72.6 Gy in this study, which was lower than the previously recommended dose of 75.6 Gy.^[18]^ However, in patients with locally advanced prostate cancer, acute urinary side effects caused by surgery or radiotherapy occurred at a similar incidence, and occurred frequently 2 to 6 months after the treatment.^[19]^ The larger volume of prostate cancer, the higher probability of developing grade 3 or higher urinary injury.^[20]^ In the present study, the tumor volume was found to be significantly smaller with small target volumes than with RTOG-defined target volumes, and therefore, patients should theoretically experience less toxic injury with small target volumes compared with the previous studies,^[19,20]^ which was confirmed by the follow-up results. Acute toxicity is an independent prognostic factor for delayed toxicity.^[21]^ Michalski et al^[22]^ reported that only 9.7% of patients with prostate cancer receiving IMRT treatment experienced grade 2 or higher gastrointestinal reactions and/or genitourinary toxicity according to the NCIC toxicity criteria. Although patients enrolled in this study to receive small target volume IMRT most had T3 or higher tumors, which increased the radiation target volume, only 4 patients experienced grade 3 or higher acute urinary injury, and 2 experienced grade 3 or higher acute intestinal reactions during the follow-up, accounting for 6.4%, which was superior to previously reported findings. Radiotherapy was able to relieve urinary symptoms in some patients,^[23]^ but the increase in radiation dose to bladder, especially trigone and neck of bladder, could significantly increase delayed urinary toxicity.^[24]^ We found a decreased radiation dose to bladder with the small target volume IMRT, which was confirmed by the fact that chronic urinary symptoms were mainly grade 2 or lower during the 5-year follow-up.

Recent studies on prognostic factors related to prostate cancer were mainly conducted at the molecular level, while clinically relevant factors have been rarely investigated.^[25,26]^ We found that physical status score was associated with prognosis in prostate cancer, and age, PSA level, Gleason score, and volume of CTV were also independent risk factors for disease progression and mortality. This may be determined by the biological characteristics of prostate cancer. The treatment of prostate cancer is closely related to hormone secretion.

However, there were many defects in our study. The design of research was not rigorous; meanwhile, the patients from 1 center could influence the results.

## 5 Conclusion

In summary, reduced-volume IMRT for prostate cancer can reduce acute and chronic injuries caused by radiation, particularly grade 3 or higher urinary and intestinal injuries, while ensuring survival benefits and protecting the hematopoietic function. However, because this was a small-sample prospective study, the sample size needs to be increased to further guide clinical treatment.

## Acknowledgments

The authors thank all the survey respondents who participated in the study and the survey field staff who did the interviews. The authors acknowledge institution that were providing administrative and logistical support to data collection: FuZhou General Hospital and the cancer organization of FuJian Province. The authors thank Jian-Hua Wu, Na Li for promoting the survey and Xue Nong, Ou Yang, and Yong-Hai Peng for many helpful suggestions.
